# Controllable and reusable seesaw circuit based on nicking endonucleases

**DOI:** 10.1186/s12951-024-02388-6

**Published:** 2024-04-01

**Authors:** Yuheng Liao, Yizhou Liu, Huan Liu, Xiao Liu, Longjie Li, Xianjin Xiao

**Affiliations:** 1 Insititute of Reproductive Health, Tongji Medical College, Huazhong University of Science and Technology, Wuhan, 430030 Hubei China; 2 School of Life Science and Technology, Wuhan Polytechnic University, Wuhan, 430023 Hubei China

**Keywords:** DNA, Seesaw circuit, Logic gate, Reusable, Controllable

## Abstract

Seesaw circuits are essential for molecular computing and biosensing. However, a notable limitation of seesaw circuits lies in the irreversible depletion of components, precluding the attainment of system recovery and rendering nucleic acid circuits non-reusable. We developed a brand-new method for creating controllable and reusable seesaw circuits. By using the nicking endonucleases Nt.BbvCI and Nt.Alwi, we removed “functional components” while keeping the “skeletal components” for recurrent usage. T-inputs were introduced, increasing the signal-to-noise ratio of AND logic from 2.68 to 11.33 and demonstrating compatibility. We identified the logic switching feature and verified that it does not impair circuit performance. We also built intricate logic circuits, such as OR-AND gate, to demonstrate the versatility of our methodology. This controllable reusability extends the applications of nanotechnology and bioengineering, enhancing the practicality and efficiency of these circuits across various domains.

**Supplementary Information:**

The online version contains supplementary material available at 10.1186/s12951-024-02388-6.

## Introduction

Watson and Crick’s elucidation of nucleic acid base pairing led to the realization of the orthogonality of DNA strands. This foundational property underpins the malleability and predictability of toehold-mediated strand displacement. This reaction serves as a cornerstone in various applications, including mutation detection [1–5], regulation and surveillance of biological behaviors [6–11], scaffold structuring [12, 13], DNA nanomachinery [14–18], coupling with gold nanoparticles [19–23], and implementation within DNA circuits [24–28].

Following Winfree’s seminal work in 2011 [29], where he introduced an amplified signal circuit (referred to as the “seesaw circuit”) based on DNA strand displacement, the field has borne witness to the development of intricate and well-designed nucleic acid networks. These networks have been artfully designed to facilitate logical operations and the fine-tuning of nucleic acid/protein interactions. Within the present landscape, two primary archetypes of nucleic acid circuits have emerged based on the driving forces behind their reactions: entropy-driven DNA cascades [29–31] and enzyme-dependent DNA catalytic systems [24, 32, 33]. Among these, the entropy-driven DNA cascade system, epitomized by the seesaw circuit, boasts a trifecta of advantages encompassing modularity, signal amplification, and robust scalability. Researchers have adeptly manipulated the structure and count of strands, as well as the configuration of toehold regions, within seesaw circuits to bolster performance attributes. These enhancements translate to swifter reaction kinetics and mitigated instances of unintended reactions, thus fortifying the circuit’s reliability [30, 34].

However, a notable limitation of seesaw circuits lies in the irreversible depletion of components, precluding the attainment of system recovery and rendering nucleic acid circuits non-reusable. This phenomenon imposes a constraint on the practical applications of seesaw circuits. Researchers have recognized this drawback pertaining to the inability to reuse seesaw circuits, prompting concerted efforts to address this issue. Regrettably, some investigations have focused exclusively on reusing singular component, whether it be the Fuel or Gate component [35–39]. Other studies necessitate intricate modifications, such as the incorporation of ultraviolet lamps into detection instruments to exert control over nucleic acid network behavior through the manipulation of light-sensitive protein structures [40, 41]. This operational adjustment entails manual intervention in regulating the activation or deactivation of ultraviolet light, resulting in a cumbersome procedure. Furthermore, the nuanced implications of these structural modifications on fluorescence signal detection remain inadequately understood. Presently, the imperative remains to establish a straightforward, viable, and controllable strategy to enable the reuse of entire DNA seesaw circuit.

Addressing this scientific issue, we have engineered a pioneering solution in the form of a reusable system (reusable circuit), hinging on the manipulation of nicking endonucleases. This innovative approach empowers us to exert artificial control over the circuit’s behavior. Unlike its reusable counterpart, this circuit possesses the remarkable capability to restore the complete nucleic acid system to its original state, rather than a partial revival, thereby enabling multiple operational cycles while ensuring robust recovery. Importantly, the reusable circuits exhibit a high degree of compatibility, as the optimized structures derived from conventional seesaw circuits can seamlessly transition to the realm of reusable circuits without the need for substantial modification.

## Results

### Principle elaboration

**Fig. 1:**
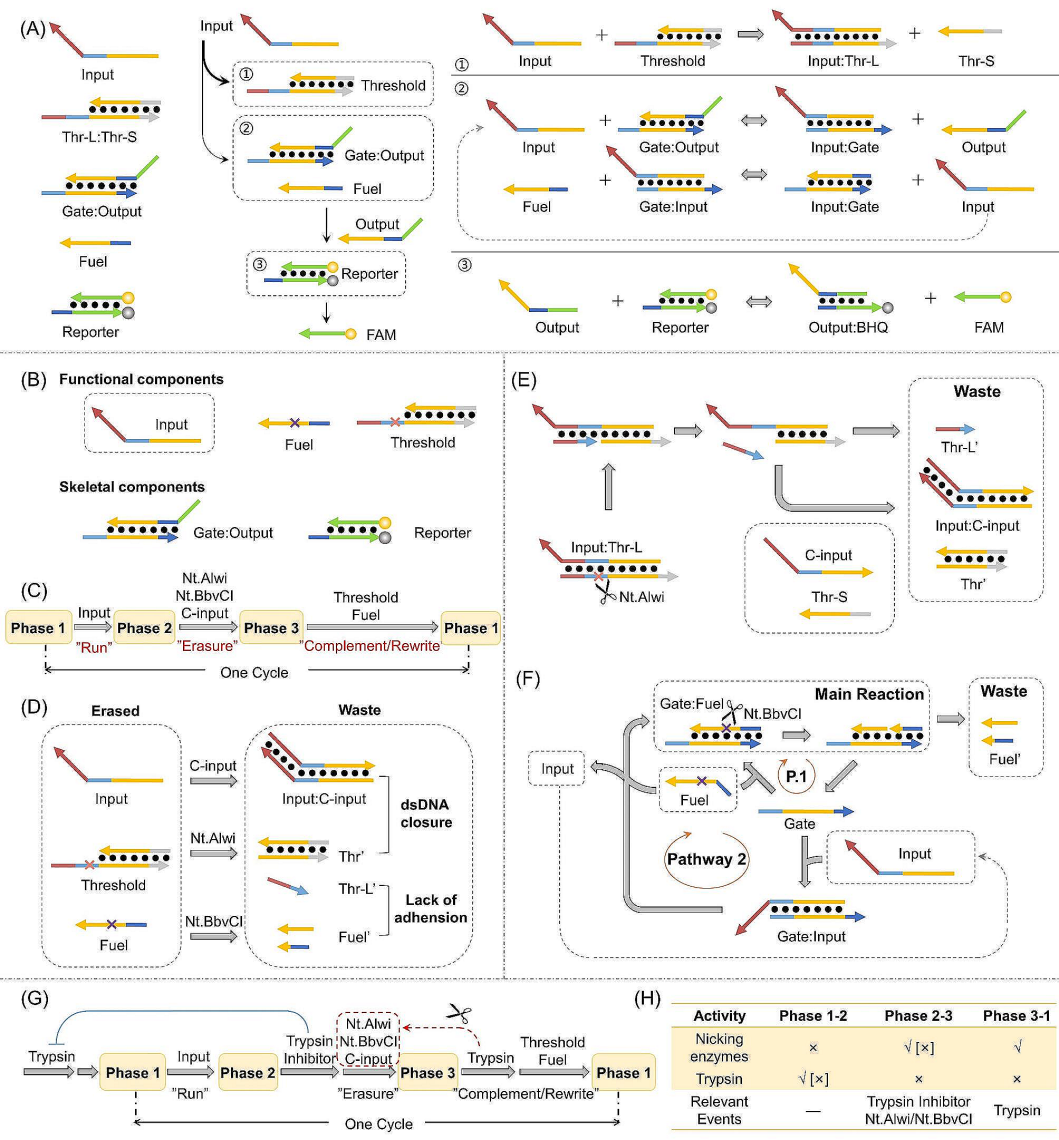
Schematic illustration of the components and working principle of the reusable circuit. (**A**) The components and logical operations of traditional seesaw circuits; (**B**) The components of reusable circuit; (**C**) Brief process of reuse; (**D**) Overview of the erasing process; working principle of erasing (**E**) Threshold and (**F**) Fuel; (**G**) The detailed process of reuse; (**H**) The introduction of enzyme activity, [ ] represent the situation during the first logic operation

The construction of the reusable circuit draws its foundation from the traditional seesaw circuit (Fig. 1A). The classical seesaw configuration encompasses essential components such as the Threshold duplex, Gate duplex (Gate: Output), Fuel strand, and Reporter duplex. Upon introduction of the Input strand, the logical reaction unfolds in a sequential triadic process. In the initial step, the Input strand preferentially interacts with the Threshold through thermodynamic and kinetic preferences. This interaction yields the formation of the Input: Thr-L duplex, effectively rendering the Input strand unavailable for subsequent stages. Subsequently, with the complete binding of the Threshold, any surplus Input strand engages with the Gate, resulting in the generation of the Output strand. Simultaneously, the formed Input: Gate duplex embarks on a reaction with the Fuel, triggering the regeneration of Input and enabling its participation in the reaction cycle, thereby facilitating Output strand production. In the final step, the free Output strand engages with the Reporter duplex, prompting the displacement of the fluorescence group FAM from its quenching counterpart BHQ. This displacement culminates in the emission of fluorescence signals, which are subsequently harnessed for detection purposes.

Expanding upon traditional seesaw circuit architecture, we innovatively introduced enzyme nicking sites within Threshold and Fuel strands to create a reusable circuit (Fig. 1B). Our approach was guided by a comprehensive understanding of seesaw circuits, leading us to categorize their elements as “skeletal components” and “functional components” (Fig. 1B).

“Skeletal components” include Gate: Output and Reporter elements, forming the circuit’s foundational framework without actively partaking in logical operations. Introduction of the Input strand initiates strand displacement reactions, converting input to output while emitting fluorescent signals through FAM labeling. “Functional components” consist of Input, Threshold, and Fuel elements, bestowing logical operation functions upon the circuit. The Input strand serves as the operational switch, commencing circuit activity. The Threshold duplex dictates logical operations, preventing leakage and ensuring multi-layered circuit stability. Fuel amplifies signals, closely tied to the Gate: Output duplex function.

The comprehensive operational cycle of the reusable circuit comprises three distinct phases and three intermediary processes (Fig. 1C). Phase 1 signifies the circuit’s initial state, serving as the starting point for logical operations. Upon introduction of the Input strand, logical processes are initiated, propelling the circuit into Phase 2. This phase signifies the completion of logical operations and the emission of fluorescent signals. Components tied to the Fuel function are omitted from the depiction for clarity. Subsequent to these logical operations, Phase 3 marks the “erasing” stage, accomplished by the addition of both the C-input strand (complementary to the Input strand) and nicking endonucleases. This erasure process ensures the comprehensive elimination of the “functional components.” In this post-erasure state, the circuit is restored to its foundational framework, featuring fully reinstated “skeletal components” while the “functional components” are expunged. The resulting byproduct termed “Waste” ceases to partake in any subsequent reactions. Following this erasure, the circuit is primed for the initiation of a new cycle. By reintroducing the “functional components,” the circuit is fully reconstituted, marking the completion of a single operational cycle. The explanations are visually represented in Supplementary Fig. 1. Importantly, it is worth noting that the “functional components” added after the erasure process need not mirror those present in the initial state. This design flexibility facilitates tailored adjustments to the reusable circuit. For instance, incorporating differing concentrations of the Threshold can recalibrate the circuit’s logic, while varying levels of Fuel can finely modulate the resulting fluorescent signals.

The fundamental concept underpinning the reusable circuit involves the erasure of the “functional components”, specifically, the Input, Fuel, and Threshold strands (Fig. 1D). Threshold erasure employs the Nt. AlwI nicking endonucleases (Fig. 1E). And the Fuel erasure is accomplished using the Nt.BbvCI (Fig. 1F). Meanwhile, the recovery of Gate: Output and Reporter are schematic illustrated in Supplementary Fig. 2.

A critical step before commencing a new operational cycle is deactivating the nicking endonucleases to prevent unintended cleavage of newly introduced Threshold and Fuel strands. We achieve this with trypsin as the inactivating agent, replenishing and resetting the system after Phase 3. After Phase 2, introducing a trypsin inhibitor is essential to inhibit residual trypsin from the previous cycle, preserving the nicking endonucleases’ integrity. Trypsin inhibitors bind specifically to trypsin’s functional sites, rendering it inactive without cleavage. Excessive trypsin, however, can overcome inhibition, restoring cleavage activity. Adjusting trypsin concentration offers precise control. This strategic approach allows reusable circuits to endure multiple cycles, ensuring sustained performance.

The comprehensive cycling process, along with the requisite substances for each phase, is visually illustrated in Fig. 1G. In Phase 1, the initiation of the operation commences with the introduction of the Input strand. Transitioning to Phase 2, the addition of a trypsin inhibitor effectively neutralizes the impact of residual trypsin from the prior cycle. This preventive measure safeguards the newly added nicking endonucleases against deactivation. The cooperative action of the nicking endonucleases and the C-input strand orchestrates the erasure of the “functional components.” Proceeding to Phase 3, during which the “functional components” are deactivated and the “skeletal components” are reinstated, the introduction of trypsin serves to inactivate the nicking endonucleases. This step forestalls the subsequent cleavage of “functional components.” Upon the reintroduction of the “functional components,” the system regresses to Phase 1, thus initiating the cycle anew. Subsequent experimental iterations adhere to this cyclical process. The roles and activities of the nicking endonucleases, trypsin, and associated enzymatic events within each phase are enumerated in Fig. 1H. It is worth noting that there is no trypsin from the previous cycle in the first run, rendering the trypsin inhibitor unnecessary.

### Demonstration of enzyme interactions

**Fig. 2:**
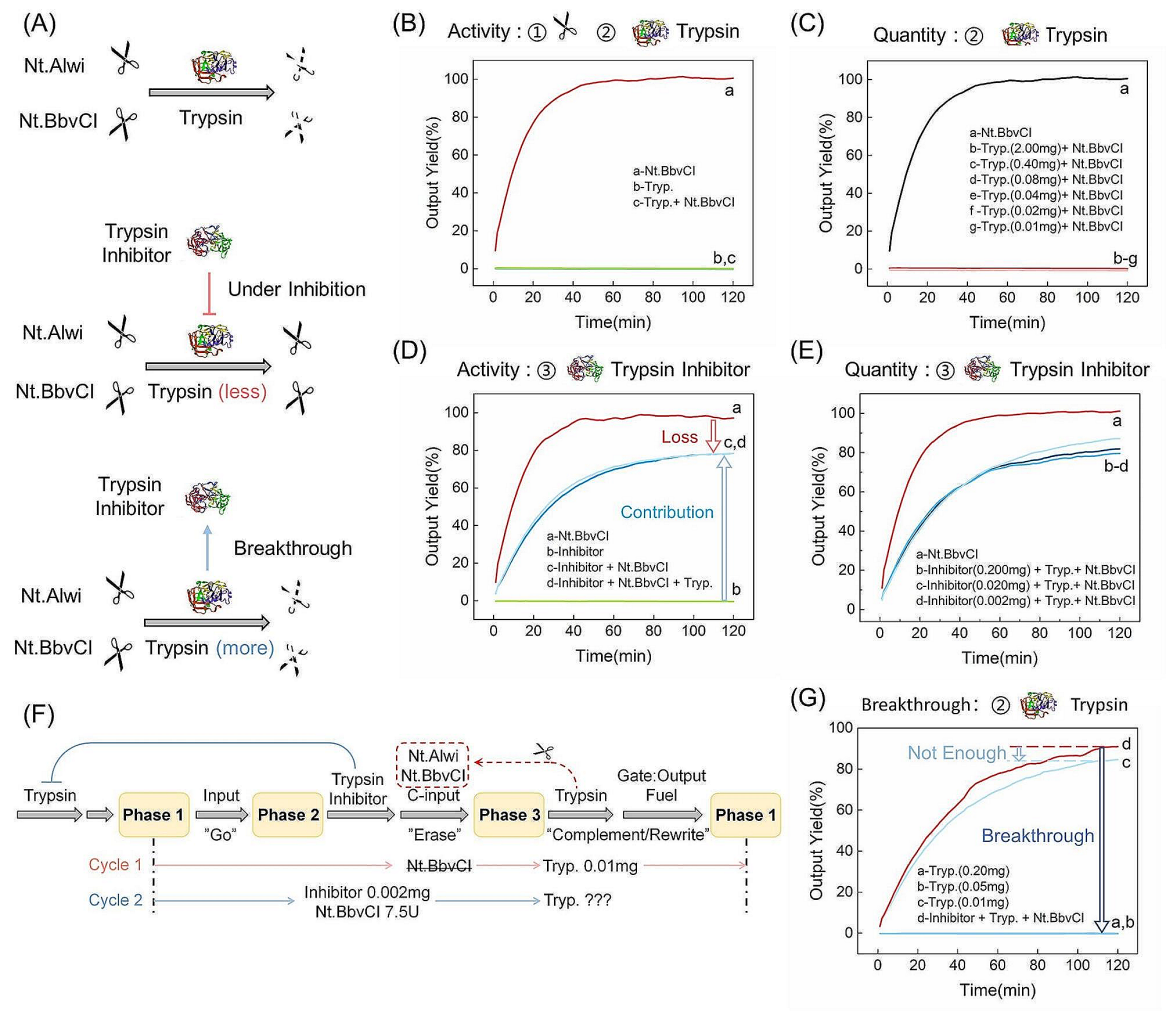
Schematic illustration of the enzymes’ function, including nicking endonuclease Nt.BbvCI, trypsin, and trypsin inhibitor. (**A**) Schematic illustration of the activity of nicking endonuclease, trypsin, and trypsin inhibitor; (**B**) Fluorescence curves of experiments demonstrating the activity of nicking endonuclease and trypsin; (**C**) Fluorescence curves of experiments with various concentrations of trypsin; (**D**) Fluorescence curves of experiments demonstrating the activity of trypsin inhibitor; (**E**) Fluorescence curves of experiments with various concentrations of trypsin inhibitors; (**F**) Experimental process and (**G**) Fluorescence curves of experiments demonstrating the breakthrough of trypsin inhibitors. Ordaining fluorescent results of fluorescently FAM-labeled strand as 100% and Reporter duplex as 0%. The nucleic acid sequence and concentration of this part’s experiments are shown in Table S1

As illustrated in Fig. 2A, we executed a comprehensive suite of experiments encompassing activity validation, interaction assessments, and concentration exploration involving three enzyme categories: nicking endonucleases, trypsin, and trypsin inhibitors. Using Nt.BbvCI as an illustrative example, it is worth highlighting the analogous rationale employed for Nt.AlwI yielded identical results, as depicted in Figure S3.

In our investigation, we validated the endonuclease activity of Nt.BbvCI through an experiment involving a designed Reporter component which can be cleaved by both Nt.Alwi and Nt.BbvCI. As shown in Fig. 2B, following the introduction of Nt.BbvCI alone, a conspicuous trend emerges. Over a 60-minute timeframe, the fluorescence signal exhibits a progressive ascent, nearing a saturation point at nearly 100%. This phenomenon underscores the successful cleavage of all FAM-labeled strands.

Sustaining the reusable circuit’s operation relies on a precise sequence of steps. To reset the system, trypsin is introduced, catalyzing the hydrolysis of nicking endonucleases. This preemptive measure prevents nicking endonucleases from inadvertently damaging the newly added “functional components,” ensuring the circuit’s ability to perform logical computations effectively. When trypsin is introduced alone, a consistent low-level fluorescence signal, approximately 0%, is maintained. This observation indicates that trypsin has no noticeable impact on the Reporter duplex or its associated fluorescence groups.

In the presence of both trypsin and nicking endonucleases, trypsin hydrolyzes the nicking endonucleases, preventing them from cleaving the FAM-labeled strand. As a result, no fluorescence signal is generated. These results align with expectations, validating the interplay of enzymes within the system and affirming their role in facilitating the operational dynamics of the reusable circuit.

Subsequently, we embarked on a series of experiments involving diverse trypsin concentrations, with the system volume consistently maintained at 50 µL and Nt.BbvCI at 7.5 U, as illustrated in Fig. 2C. Within the specified trypsin dosage range spanning from 2 mg to 0.01 mg, the fluorescence signal remains consistently low. This outcome establishes that the catalytic activity of the nicking endonuclease Nt.BbvCI is effectively neutralized through complete hydrolysis. In following tests, 7.5U of Nt.BbvCi were hydrolyzed using 0.01 mg of trypsin powder.

To render the “functional components” inactive after one or more cycles in Phase 2, we add nicking endonucleases. However, these enzymes are hydrolyzed by trypsin in the subsequent cycle, necessitating the addition of trypsin inhibitors. We then systematically assessed the trypsin inhibitors’ efficacy in deactivating trypsin (Fig. 2D). The fluorescent signal did not increase when both Reporter and inhibitor were present (Curve b), confirming that trypsin inhibitors did not nick the Reporter. When Nt.BbvCI and trypsin inhibitor were injected concurrently (Curve c), the fluorescent curve increased to a high level but dipped somewhat lower than Curve a, indicating that the nicking endonuclease could still function but with reduced activity in the presence of trypsin inhibitor. This effect may be due to increased inhibitor concentration reducing binding interactions between the nicking endonuclease and the target strand or marginal inhibition of the nicking endonuclease’s catalytic activity. Furthermore, with trypsin inhibitors present, the introduction of trypsin no longer affected the fluorescence signal (Curve c and d overlapped), confirming comprehensive trypsin deactivation.

In optimizing the trypsin inhibitor concentration, we explored various concentrations: 0.2 mg, 0.02 mg, and 0.002 mg. All three concentrations resulted in nearly identical fluorescence curves (Fig. 2E). This finding indicates that even the minimal 0.002 mg dosage of trypsin inhibitor effectively deactivates 0.01 mg of trypsin. Lower concentrations were not tested.

Trypsin inhibitors, with their exclusive role in trypsin deactivation and no nicking capacity, are pivotal to the system. Using an excessive quantity of trypsin can overcome the inhibitor’s effect. Therefore, a nuanced strategy emerges. Even after completing multiple operational cycles, the addition of trypsin remains an option. This strategic introduction catalyzes the hydrolysis of the nicking endonuclease, resetting the system for further operation.

To mimic the state of enzymes within the system after a single cycle and prepare for trypsin reintroduction, we conducted a simulation-based experiment (Fig. 2F). It is essential to note that nicking endonuclease initially introduced has been proven to be hydrolyzed by trypsin in prior experiments and is not reintroduced for subsequent cycles. Upon adding 0.01 mg of trypsin, a noticeable increase in fluorescence signal occurs (Fig. 2G), confirming the nicking endonuclease’s involvement in cleaving the FAM-labeled strand. However, when 0.2 mg or 0.05 mg of trypsin is introduced, there is no change in the fluorescence signal (Fig. 2G). This aligns with the Reporter group’s behavior, indicating that the FAM-labeled strand remains intact, demonstrating that trypsin overcomes the trypsin inhibitor’s blockade.

We demonstrate the controllable regulation of trypsin’s enzymatic activity in the presence of both trypsin and trypsin inhibitor. By adjusting their relative concentrations, we can effectively govern trypsin’s activity. This control mechanism enables the reusable circuit to undergo multiple cycles, ensuring accurate logical operations.

### Principle validation: OR and YES-OR logic gates

**Fig. 3:**
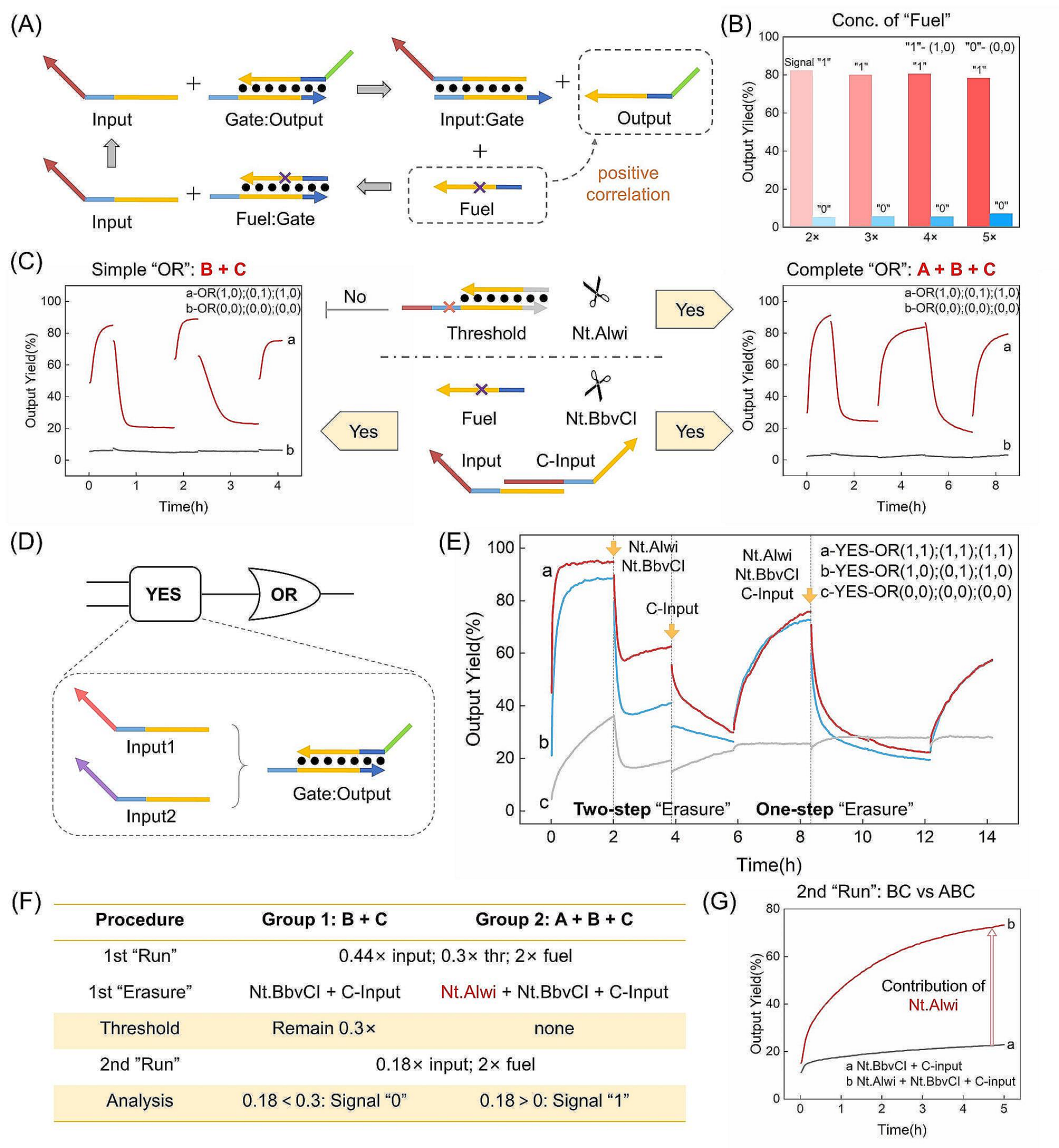
Schematic illustration of principle validation. (**A**) Schematic illustration of the amplification effect of Fuel strand; (**B**) Fluorescence results of experiments with various concentrations of Fuel strand; (**C**) Schematic illustration and fluorescence curves of three runs of simplified OR gate and complete OR gate; (**D**) Schematic illustration of the upstream YES gate in YES-OR gate; (**E**) Fluorescent curves of three runs of YES-OR gate; (**F**) Experimental process, critical points and (**G**) fluorescent curves of experiments directly validating the function of Nt.Alwi. Ordaining fluorescent results of Output + Reporter as 100% and Reporter duplex as 0%. The nucleic acid sequence and concentration are shown in Table S2. Original fluorescence curves and processing of Simple OR gate are demonstrated in Figure S4

In the seesaw circuit, the Fuel component assumes a pivotal role in the realm of signal amplification (Fig. 3A). We tried a variety of Fuel chain concentrations to achieve proper signal amplification(Fig. 3B). The outcomes reveal that when the Fuel concentration assumes values of “2×,” “3×,” “4×,” or “5×,” discernible differences elude detection in fluorescence signals. Subsequent experiments maintained a Fuel concentration of “2×.”

To demonstrate the function assumed by Nt.BbvCI and the C-input within the context of the system’s restoration process, we engineered a simplified “OR” logic gate configuration, omitting the presence of Threshold components. This configuration was subjected to a series of operational cycles, spanning three distinct runs (Fig. 3C, left). The restorative trajectory of the simplified “OR” logic gate pivots upon the introduction of Nt.BbvCI and the C-input, with Nt.Alwi’s involvement omitted. In restoration processes, the fluorescent signal consistently diminishes, eventually plateauing at approximately 20%. This conspicuous attenuation effectively validates the collaborative impact of Nt.BbvCI and the C-input.

We proceeded to construct a complete “OR” logic gate, encompassing all relevant components, and embarked on a series of three operational cycles (Fig. 3C, right). The initial operational cycle showed an impressive fluorescence signal of 92% within one hour. Subsequent iterations showed noteworthy multiplexing capabilities, with the third run reaching 80% of the fluorescence signal within an hour. The pivotal role played by Nt.Alwi, Nt.BbvCI, and the C-input was showed as these components collectively orchestrated the erasure process in which the fluorescent signal reduced to 20%. Concurrently, the leakage group maintained a consistently low fluorescence signal. These outcomes decisively affirm the fundamental feasibility of the reusable seesaw circuit.

Additionally, we created the YES-OR logic and executed a series of three operational cycles, in which the YES gate was conceived as a strand displacement reaction, as illustrated in Fig. 3D. Throughout these successive runs (Fig. 3E), the fluorescence signal demonstrated remarkable progress, achieving 60% within 2 h. Notably, the latter two runs exhibited an encouraging trajectory, with the fluorescence signal persistently displaying an upward trend even after 2 h. Following two rounds of erasure, the signal diminished to levels below 30%. In the first erasure process, the fluorescence signal decreased to 30% and continued to manifest a notable downward trend. In the subsequent erasure cycle, the signal further decreased to a lower 20%. Meanwhile, the fluorescence signal within the leakage group reached a peak of 35% during the initial run, subsequently maintaining a consistent sub-30% level. The discernible contrast between signals “1” and “0” offers a provement to the successful runs of YES-OR logic, effectively endorsing the system’s ability to consistently and accurately perform its intended logical operations.

In the first erasure process (Fig. 3E), a two-step erasure strategy was deployed. The sequential introduction of two distinct nicking enzymes (AB) preceded a subsequent addition of the C-input (C). This orchestrated sequence elicited a segmented decrease in the fluorescence signal. It is noteworthy to highlight that Nt.Alwi’s role within this context did not contribute to the decrease in the fluorescence signal. This experiment further solidified the demonstration that Nt.BbvCI and the C-input are pivotal in orchestrating the decrease of the fluorescent signal, confirming their indispensable roles. Additional explanation of Fig. 3E is provided in Supplementary Note 1.

Based on the principle analysis and experimental validation, the successful execution of the second-cycle reaction substantiates the efficacy of Nt.Alwi. For a more rigorous proof of the role of Nt.Alwi, we devised an experiment to directly show the nicking action of Nt.Alwi. As illustrated in Fig. 3F, the introduction of Nt.Alwi determines whether the second run is successful or not. During the initial run, the Input concentration exceeded that of the Threshold (0.44 > 0.3), permitting Input to surmount the threshold barrier and elicit a positive response. The second run assumes importance in directly substantiating the role of the nicking endonuclease Nt.Alwi. During this phase, both groups received “0.18×” Input and “2×” Fuel. In the control group employing the “ABC” strategy, the Threshold underwent nicking, thereby enabling the reusable circuit, now equipped with both “power” and “starter,” to initiate an operation. This translated into a rapid surge in the fluorescent signal (Fig. 3G). Conversely, the experimental group, employing the “AB” approach, maintained the Threshold’s concentration at “0.3×,” exceeding that of the introduced Input (0.3 > 0.18). It hindered the forward reaction, leading to a minimal increase in the fluorescent signal (Fig. 3G). Through these meticulously designed experiments, we have directly and conclusively verified the role of the nicking endonuclease Nt.Alwi in acting upon the Threshold.

Consequently, the roles of Nt.Alwi, Nt.BbvCI, and C-input in the erasure process of reusable circuits have been independently, directly, and meticulously demonstrated in both single-layer and double-layer circuit configurations.

### Compatibility demonstration of reusable circuits

We designed the reusable circuit with two enzyme-nicking sites, building upon the foundation of traditional seesaw circuits. With minimal adjustments, this design ensures that the reusable circuit inherits all the compatibility advantages of its traditional counterpart.

**Fig. 4:**
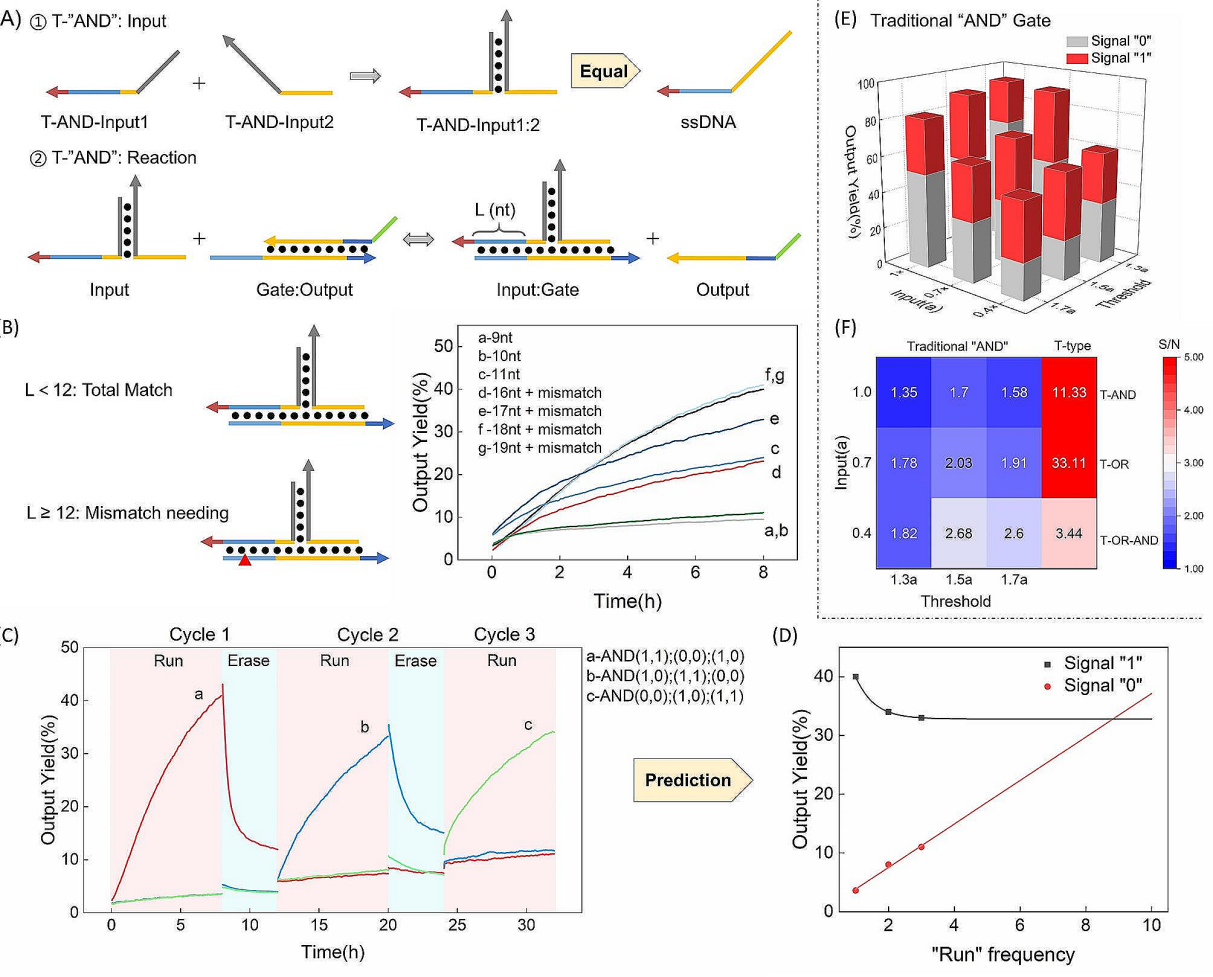
Schematic illustration of working principle and fluorescent curves of T-type input AND gate-related experiments. (**A**) Schematic illustration of working principle of T-type input gate; (**B**) The schematic illustration and fluorescent curves of T-AND gate under the regulation of different length of the T-type input’s toe; (**C**) Fluorescent curves of T-AND three-round operations; (**D**) Prediction of the number of T-AND runs; (**E**) The fluorescent results of traditional AND gate; (**F**) The signal-to-noise ratio of the traditional seesaw and T-AND. Ordaining fluorescent results of Output + Reporter as 100% and Reporter duplex as 0%. The nucleic acid sequence and concentration are shown in Table S3

For the purpose of compatibility validation, we specifically chose the T-type input AND gate (T-AND), known for its exceptional anti-leakage functionality. We conducted an investigation into the toe length of the T-AND-Input, denoted as “L” (nt) (Fig. 4A). As illustrated in Fig. 4B, we explored cases where L is less than 12, attempting values of L = 9, 10, and 11. For cases where L is greater than or equal to 12, an essential measure was to introduce one mismatch on the Gate strand to safeguard against the potential nicking of Input: Gate by the nicking endonuclease. Drawing from past experiences, compensating for the loss of binding strength due to a 1nt mismatch necessitates the incorporation of an additional 3–5 nt base pairing. Therefore, we examined the scenarios where L = 16, 17, 18, and 19. The fluorescent curves reached their peaks at L = 18/19, and minimal disparity existed between the two curves. As a result of this evaluation, we opted to proceed with L = 18 for subsequent experiments.

T-AND reuse proved successful, as evidenced by the results of three runs, as depicted in Fig. 4C. Across these runs, after 8 h of reaction, the “1” output signals registered at 40%, 33%, and 34%, while the “0” output signals stood at 3.6%, 8.0%, and 11.0%, respectively. At the 8-hour mark, the “1” signal in the second run decreased by 7% compared to the first run, while the “1” signal in the third run only decreased by 1% compared to the second. It is anticipated that the “1” signal will experience minimal or no loss in subsequent runs. Fitting the data using the exponential function yielded ${Y_{(1)}} = 32.8 + 43.2 \times {0.167^x}$. Though there was a slight decrease in the “0” signal during the erasing process, the overall trend indicated a linear increase. The “0” signal is projected to continue its linear growth in the following runs. Employing the linear function for fitting yielded ${Y_{(0)}} = 0.133 + 3.70x$. The intersection of these two functions occurs at x = 8.8, suggesting that the T-AND circuit is capable of operating correctly for 8 cycles (Fig. 4D). It is important to note that the fitting method might not be optimal due to the limited dataset of only three runs. The presented results are for illustrative purposes and might diverge from the actual scenario.

To highlight the superior signal-to-noise (S/N) ratio of the T-type input, we developed AND gates using traditional seesaw circuits (Fig. 4E). By maintaining consistent concentrations of the other system components, we obtained nine results with an S/N ratio below 3 through continuous adjustments of input and threshold concentrations. Meanwhile, both T-AND and the subsequently constructed T-OR gate exhibited S/N ratios exceeding 10, while the double-layer circuit T-OR-AND also maintained an S/N ratio above 3 (Fig. 4F). This underscores the substantial S/N ratio advantage of the T-type input gate.

### Additional performance: Logic switching and OR-AND complex circuit

**Fig. 5:**
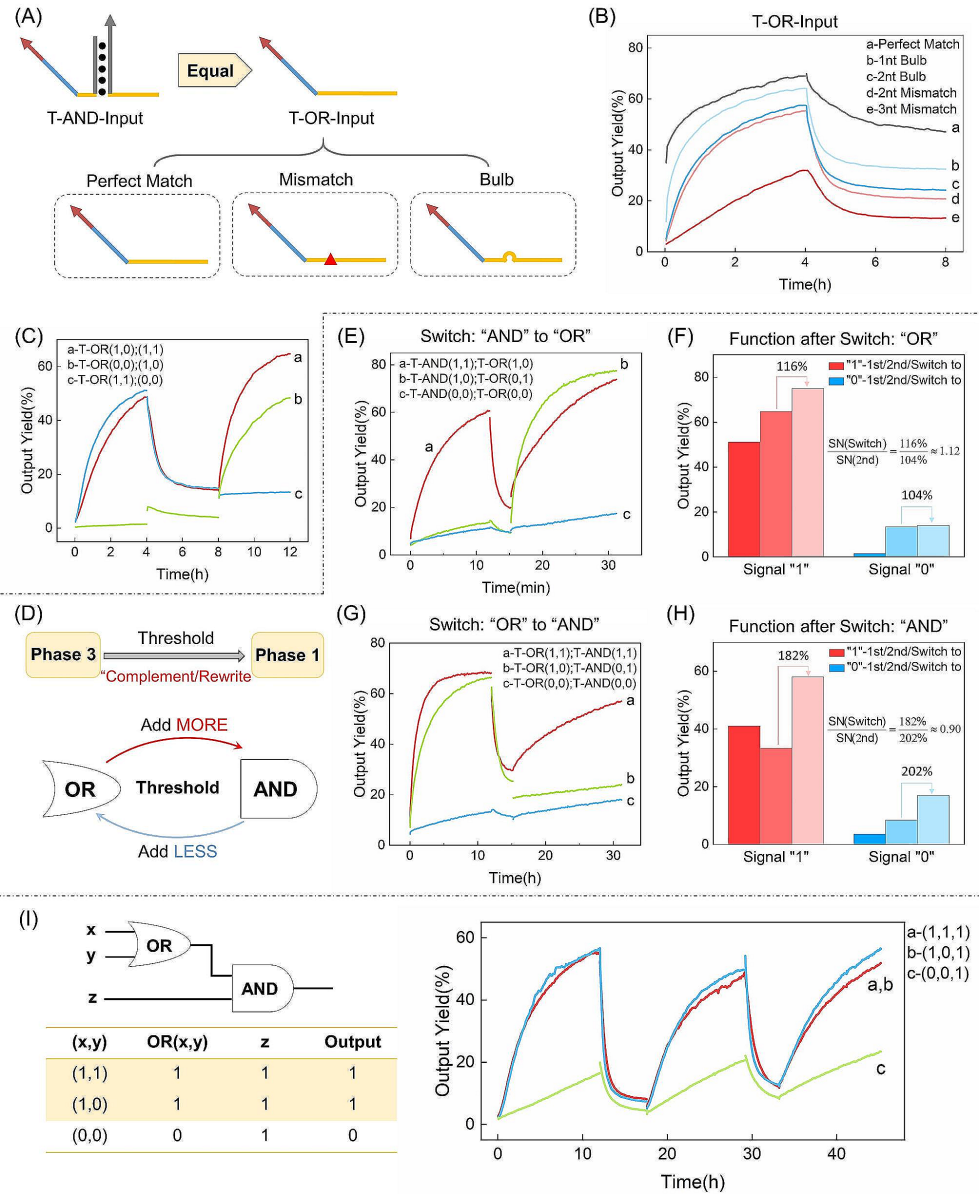
(**A**) Schematic illustration of the structure of T-OR-input; (**B**) Fluorescent curves of experiments with various structures of T-OR-input; (C) Fluorescent curves of T-OR two-round operations; (**D**)~(**H**) Schematic illustration and fluorescent curves of mutual switching between T-AND and T-OR logic circuits; (**I**) Fluorescent curves of T-OR-AND three-round operations. Ordaining fluorescent results of Output + Reporter as 100% and Reporter duplex as 0%. The nucleic acid sequence and concentration are shown in Table S4

We introduced an Input strand analogous to T-AND-Input to establish an OR gate (T-OR) (Fig. 5A). Interestingly, when T-OR Input and Gate are a perfect match, the decrease in fluorescent signal during the erasing process is insufficient. To address this issue, we introduced a mismatch or “bulb” on T-OR-Input to attenuate the binding strength of the Input: Gate duplex (Fig. 5A). Subsequently, upon decreasing the binding force of Input: Gate, the anticipated reduction in fluorescent signal during erasing occurred (Fig. 5B). We selected T-OR-Input with a 2nt mismatch for subsequent experiments. The results confirmed that T-OR could perform two correct operations (Fig. 5C). After 4 h, the “1” signal reached 50%, while the “0” signal consistently remained below 15%.

As previously mentioned, during the transition from phase 3 to phase 1, circuit logic and signal amplification efficiency can be fine-tuned by adjusting the concentrations of Fuel and Threshold (Fig. 5D). This transition entails adding a reduced amount of Thresholds, yet still yielding accurate logical outcomes (Fig. 5E). To investigate whether logic switching affects the computational power of reusable circuits, we conducted a comparison between continuously running OR gates and OR gates that were switched from AND logic. (Fig. 5F). Similarly, in the second run, the “1” signal of the OR gate increased to 116%, and the “0” signal increased to 104% after the switch, both compared to non-switching conditions. The S/N ratio post-switching was 112% of the non-switching case, affirming that logic switching does not impact the computational capacity of the OR gate. Subsequently, we compared continuously running AND gates with AND gates converted from OR gates (Fig. 5G). Following the switch, the “1” signal increased to 182%, and the “0” signal increased to 202%, both compared to the non-switching scenario. The S/N ratio post-switching was 90% of the non-switching case, demonstrating that logic switching does not compromise the AND gate’s computational power (Fig. 5H). Finally, we constructed a reusable double-layer circuit, the OR-AND configuration. Completing three runs showed the reusable circuit’s capability to construct intricate circuits (Fig. 5I).

## Discussion

Reusable sesaw circuits are built on the basis of traditional sesaw circuits. The core of its reusability lies in the erasure of “functional components”. The Input strand undergoes erasure through hybridization with its complementary counterpart, the C-input strand, thereby generating the Input: C-input duplex (Fig. 1D). In parallel, nicking endonucleases cleave the Threshold strand, resulting in the formation of two distinct segments, the Thr’ duplex and the short single-stranded Thr-L’. Similarly, the Fuel strand is subjected to cleavage, yielding two short single strands collectively termed as Fuel’. These four cleavage products become the “Waste”, rendering no subsequent reactions. Both Input: C-input and Thr’ adopt a “duplex closure” configuration, characterized by their tightly bound, toe region-lacking structure and challenging to initiate strand displacement reactions. Meanwhile, Thr-L’ and Fuel’, being short single strands fewer than 11 nucleotides, their respective duplex structures are inherently prone to spontaneous dissociation at a reaction temperature of 37 ℃.

As illustrated in Fig. 1E, Nt. AlwI cleaves the Thr-L strand within the Input: Thr-L duplex, yielding a short single-stranded fragment, which becomes the “Waste.” Subsequently, the remaining duplex is invaded by the C-input and Thr-S strands, with the C-input aligning with Input via hybridization, while Thr-L reunites with Thr-S. This forms a “duplex closure” configuration, preventing further reactions and thoroughly erasing Threshold strands.

Erasure process of Fuel involves the main reaction and two pathways (Fig. 1F). In the main reaction, Nt.BbvCI cleaves the Fuel strand within the Gate: Fuel complex, generating truncated single strands as waste. However, the circuit often contains excess Fuel, typically twice the Gate strand concentration. Relying solely on the main reaction is insufficient to fully cleave Fuel strands. Therefore, two additional pathways are crucial. In Pathway 1, the Fuel’ disengage post-cleavage, causing the dissociation of the Gate strand from the cut Fuel strand. This dissociated Gate strand then reunites with free Fuel, thus entering the main reaction anew. Furthermore, the unrestrained Gate strand has the potential to hybridize with the Input strand, thereby initiating Pathway 2. Within Pathway 2, the Gate: Input duplex reacts with Fuel, resulting in the production of the Gate: Fuel duplex, which serves as the substrate for the main reaction. Meanwhile, the Input strand disengages. Notably, the thermodynamic driving force of this reversible reaction is limited (∆ G ≈ 0), permitting about 50% of the participating Fuel molecules to hybridize with Gate and partake in the main reaction. Thereafter, the Input strand, the terminal product of Pathway 2, circulates back into the Pathway 2 as a reactant. This recycling process propels Pathway 2 forward, facilitating the complete inclusion of Fuel strands in the main reaction, leading to their erasure. Throughout the erasure of the “functional components,” the “skeletal components,” Gate: Output and Reporter, revert to their initial state as illustrated in Figure S2.

In the demonstration of enzyme interactions, we designed a Reporter duplex which can be cleaved by both Nt.Alwi and Nt.BbvCI. Initially, the isolated Reporter duplex exhibited no detectable signal due to the effective quenching mechanism between the FAM fluorescent group and BHQ1. Upon introducing Nt.BbvCI, this nicking endonuclease cleaved the FAM-labeled strand within the Reporter, generating two truncated single strands. The truncated strand containing the fluorescent group was exceptionally short, only six nucleotides long, which significantly reduced its thermal stability (Tm ≈ 0 ℃). Consequently, it could not form a stable duplex structure at the experimental temperature of 37 ℃. This inability to form a duplex effectively separated the FAM fluorescent group from the quenching moiety, resulting in the emission of a fluorescence signal.

In the demonstration of principle, we categorize Nt.Alwi, Nt.BbvCI, and C-input into two groups according to their different functions. Nt.BbvCI and C-input collaborate to erase the Fuel and Input strands, depleting the “power” and “starter” components and transitioning the circuit from phase 2 to phase 3, evident in the diminishing fluorescent curve. Nt.Alwi’s role lies in erasing the Threshold duplex. While this may not directly impact the fluorescent signal, it is pivotal for logical circuit. Failure to erase the Threshold would hinder the circuit’s ability to perform logical operations in subsequent cycles. As a result, during an operational cycle, the diminishing fluorescent signal in phases 2 and 3 is influenced by Nt.BbvCI and C-input. However, when preparing for another cycle, the successful execution of logical operations from phase 1 to phase 2 confirms Nt.Alwi’s effectiveness.

Subsequently, we demonstrate the compatibility of reusable circuits using the T-AND circuit. The T-AND circuit differs from traditional seesaw circuits primarily in terms of input handling, as the fundamental structure of other components remains unchanged. In the T-AND configuration (Fig. 3A), the input state is dictated by the type of input added. Adding no input signifies an input state of (0, 0) and leads to an output of 0. Adding one type of Input denotes an input state of either (1, 0) or (0, 1), both resulting in an output of 0. Introducing both two types of Input signifies an input state of (1, 1) and yields an output of 1. By combining the two Input strands, a T-type input (T-Input1:2) is formed, complete with ample invasion and dissociation domains. This T-type input can interact with the Gate component to generate the Output, ultimately culminating in the emission of fluorescent signals. The design principles for the reusable circuit remain consistent with those outlined earlier. The T-AND circuit was designed with nicking sites on each Fuel and Threshold component. Following the established principles and experimental procedures, the T-AND circuit demonstrates its reusability.

At the beginning of the construction of T-OR circuit, we found that the fluorescence signal degradation during erasure was not as expected. This phenomenon is attributed to the much stronger binding force between T-OR-Input and Gate strands compared to T-AND-Input owing to high resistance at the triple strand turning point of T-AND-Input: Gate duplex. It led to the Gate’s preferentially binding to Input during erasing in T-AND. As depicted in Fig. 1F, when pathway 1 weakens, pathway 2 stalls in the Input: Gate state, leading to reduced Fuel: Gate generation and incomplete erasure of the Fuel strand.

In conclusion, we have successfully designed a controllable reusable circuit based on nicking endonucleases. This system enables the repeated use of seesaw circuits under constant temperature conditions (37 ℃) and exhibits timely signal output responses. Our experiments have not only validated the feasibility of reusable circuits but also showcased the ability for multiple reuses. Additionally, using the T-input as an illustrative example, we highlighted the robust compatibility and logic switching capabilities of reusable circuits. Furthermore, by constructing an OR-AND logic circuit, we demonstrated the system’s capability to build complex circuits. However, it is important to note that the reusable circuits still require further optimization. As multiple runs are completed, the accumulation of waste products may impede the speed and effectiveness of logical operations, potentially leading to a breakdown of the DNA network (Supplementary Note 2). we have explored solutions to this limitation as follows.

1. Two types of nicking endonucleases were used in order to cut the Fuel and Threshold components in this study. By unifying the sequences of their cleavage sites, it is possible to use only one nicking endonuclease for cleavage to reduce enzyme usage and increase the number of reusable times. Theoretically, unifying the sequences of cleavage sites would have an impact on the computing power. Therefore, we finally adopted the design in the paper with two kinds of nicking endonucleases, sacrificing the number of reusable times to a certain extent, in exchange for the expandability of reusable circuits. At the same time, we also noted that Winfree’s study of seesaw circuits published in 2011 made extensive use of the same sequence of toe regions, but the logic circuits in that study still showed strong arithmetic capabilities. It can be speculated that a more refined sequence design in reusable circuits could allow Fuel and Threshold to unify the enzyme cleavage sites and reduce the use of nicking endonuclease to increase the number of reuses.
2. In this study, all circuits consist of DNA strands. Compared to DNA strands, RNA strands are simpler to cut, and RNA enzymes can cut RNA strands of any sequence into 3nt fragments. Designing the Fuel and Thr-L in the reusable circuits as RNA strands not only reduces the use of enzymes (RNA enzymes can cut Fuel and Thr-L simultaneously), but also reduces the effect of “waste” in the circuits (3nt fragments are almost unable to participate in or affect the chain substitution reaction at 37 °C). However, due to the lack of experience in the construction of DNA-RNA circuits, we did not use this option.

Meanwhile, the use of nicking endonucleases will impose limitations on the design of partial sequences. The limitation typically affects 4-6nt DNA sequences. However, the impact of this limitation on the ability of reusable circuits is substantially less than we expected. There are hundreds of different types of nicking endonucleases. We can access to 106 “endonuclease” from NEB and 176 from Thermo Fisher. A wide range of direct-orderable nicking endonucleases allowed us to design the sequences with little or no impact. Despite these, our research addresses a significant gap in the field by establishing the reusability of seesaw circuits. The controllable reusability we have achieved will enhance the potential application scope of seesaw circuits in areas such as nanomachines and biological regulation. From our perspective, reusable circuits have potential applications in mutation detection, DNA nanomachines and nucleic acid framework regulation. Nucleic acid circuits possess signal amplification modules that can effectively improve the detection limit of mutation detection. Simultaneously, the construction process of the microsystem required for mutation detection is cumbersome. The introduction of reusability can avoid building the system from scratch and shorten the preparation time for mutation detection. Similar to the logic switching function of reusable circuits, the introduction of reusability may be able to controllably switch the behavior and function of DNA nanomachines. By designing different functions as modular “plug-ins”, it not only reduces the difficulty of designing nanomachines, but also gives them diverse functions. The idea of reusable circuits can also be applied to the regulation of nucleic acid frameworks. The complete nucleic acid framework system can be unitized, and the entropy of the system can be regulated by erasing and supplementing the connecting elements of the unit, thus achieving the switching between the nucleic acid solution and the nucleic acid framework at the macroscopic level. The continuous advancement of this field will likely involve the design of more high-performance reusable strategies to further drive its development.

## Materials and methods

### Materials

Oligonucleotides used were purchased from Sangon Biotech (Shanghai). Oligonucleotides were checked by Sangon Biotech by capillary electrophoresis for purity and by electrospray ionization mass spectrometry for identity. All labeled strands (FAM and BHQ-1) were HPLC purified by Sangon Biotech. Oligonucleotides were ordered in the dry format and suspended in Tris-EDTA buffer. Concentrations were determined by measuring the absorbance at 260 nm using a NanoDrop 2000 spectrophotometer (Thermo Fisher Scientific).

Nicking endonucleases Nt.Alwi and Nt.BbvCI (Both are nicking endonuclease that cleaves only one strand of the double-stranded DNA substrate. Nt.BbvCI recognizes the sequence CC∧TCAGC. Nt.Alwi recognizes the sequence GGATCNNNN∧N), CutSmart buffer (50 mM potassium acetate, 20 mM Tris-acetate, 10 mM magnesium acetate, and 100 µg/mL BSA, pH 7.9 at 25 ℃), ThermoPol reaction buffer (20 mM Tris-HCl, 10 mM KCl, 10 mM (NH_4_)_2_SO_4_, 2 mM MgSO_4_, and 0.1% Triton X-100, pH 8.8 at 25 ℃) were purchased from New England Biolabs.

### Methods

#### DNA sequence design

DNA sequences were designed using the NUPACK server (http://www.nupack.org) and the OligoAnalyzer Tool (https://www.idtdna.com/pages/tools/oligoanalyzer).

#### DNA duplex preparation

Strands forming the DNA duplex were mixed in an experimental buffer. Each 50 µL aliquot of the DNA duplex was heated up to 95 °C for 5 min and then cooled to room temperature over 2 h. Strands that formed reporter complexes were annealed in an experimental buffer with a 5% excess of the quencher-labeled strand.

#### Fluorescence data analysis

Data acquired by the Gen5 software was exported to a Microsoft Excel file, which was subsequently imported, analyzed, and plotted using ORIGIN software. Time was linearly adjusted so that t = 0 corresponds to the first data point acquired after the plate strips were put into the machine after the addition of reagents. The data analysis of reusable circuits involves normalization and integration of multiple curves, which are show in Figure S4.

### Electronic supplementary material

Below is the link to the electronic supplementary material.

Supplementary Material 1

## Data Availability

The datasets used and/or analyzed during the current study are available from the corresponding author upon reasonable request.
