# Supplementary material for: Controllable and reusable seesaw circuit based on nicking endonucleases
Source: J Nanobiotechnology. 2024 Apr 1;22:142. doi: 10.1186/s12951-024-02388-6 (PMC10983715; doi:10.1186/s12951-024-02388-6)
Supplement: Supplementary file 1 — Supplementary Material 1 [file 12951_2024_2388_MOESM1_ESM.docx]

**Supplementary Information for**

**Controllable and Reusable Seesaw Circuits Enabled by Nicking Enzymes**

**Supplementary Note 1. Additional explanations of three runs of YES-OR gate.**

There are three key observations to underscore regarding the fluorescent curves generated in three runs of YES-OR gate:

1. The Initial Erasing Process: When the initial erasing process introducted two nicking enzymes, Curve a exhibited a decrease of approximately 60%, while Curve b exhibited a decrease of around 40%. This discrepancy finds its origin in the distinct contributions made by Fuel and Input to the fluorescent signal. Within the logical operations, the (1, 1) group (Curve a) introduced a greater quantity of Input strand as compared to the (1, 0) group (Curve b). Consequently, the contribution of Fuel to the signal is relatively minor, translating to a comparatively modest reduction in signal post-erasure.

2. An Intriguing Phenomenon: The fluorescence curve experienced a rapid decrease, followed by an inflection point and gradual ascent in the first erasure process illustrated by Curve a, b. This could potentially be attributed to the swift initial pace of enzymatic nicking. Subsequently, as Fuel became depleted, the Reporter duplex swiftly reformed, leading to an apparent initial decline in the fluorescence signal. Subsequent to the completion or deceleration of the enzymolysis process due to substrate exhaustion, the system converged toward equilibrium. This, in turn, induced partial dissociation of the Reporter duplex, resulting in a gradual elevation of the fluorescence signal.

3. Fluorescent Signal Restoration and (0, 0) Group Analysis: During the erasure process, the (0, 0) group did not involve the addition of C-input but incorporated two nicking enzymes. In theory, the (0, 0) group does not emit a fluorescent signal, rendering the inclusion of additional components unnecessary during the erasure phase. However, for practical ease and uniformity in operational protocols, we incorporated two types of nicking enzymes across all groups during the erasure process. This standardization permits personnel who may not be entirely familiar with the underlying principles of the reusable circuit to execute the procedure correctly. Concurrently, the (0, 0) group generated a minimal degree of leakage throughout its operation, yielding a low fluorescent signal. This discrepancy predominantly arises from the upstream YES gate's Gate component (Gate: Output duplex) invading the toe-free zone of the downstream OR gate's Gate component. This interaction engenders a leakage reaction driven by relatively weak thermodynamic and kinetic forces (Figure 3, E). Notably, the (0, 0) group (Curve c) exhibits a decline in the fluorescent signal during the erasure process, thereby signifying the potential for rectification of the leakage reaction. Given the minute scale of the leakage, its impact on the functional components remains marginal. Consequently, it exerts negligible influence on subsequent logical operations, rendering the reintroduction of "functional components" unnecessary.

**Supplementary Note 2. Limitations of reusable circuits-1: Limitations of the number of reuses.**

The application of multiple enzymes and the presence of “waste” will inevitably pose challenges to the arithmetic capacity and number of reuses of the reusable circuit. We recognized this limitation in project design and analysis of experimental results. In fact, my colleagues and I agreed that the importance of this research lies in giving traditional seesaw circuits an artificially controlled reusability, while fully retaining the advantages of modularity, scalability, and compatibility, rather than the number of reuses. As a consequence, we have focused more on the proof of reuse principle for reusable circuits than on the number of times a particular logic gate can be reused.


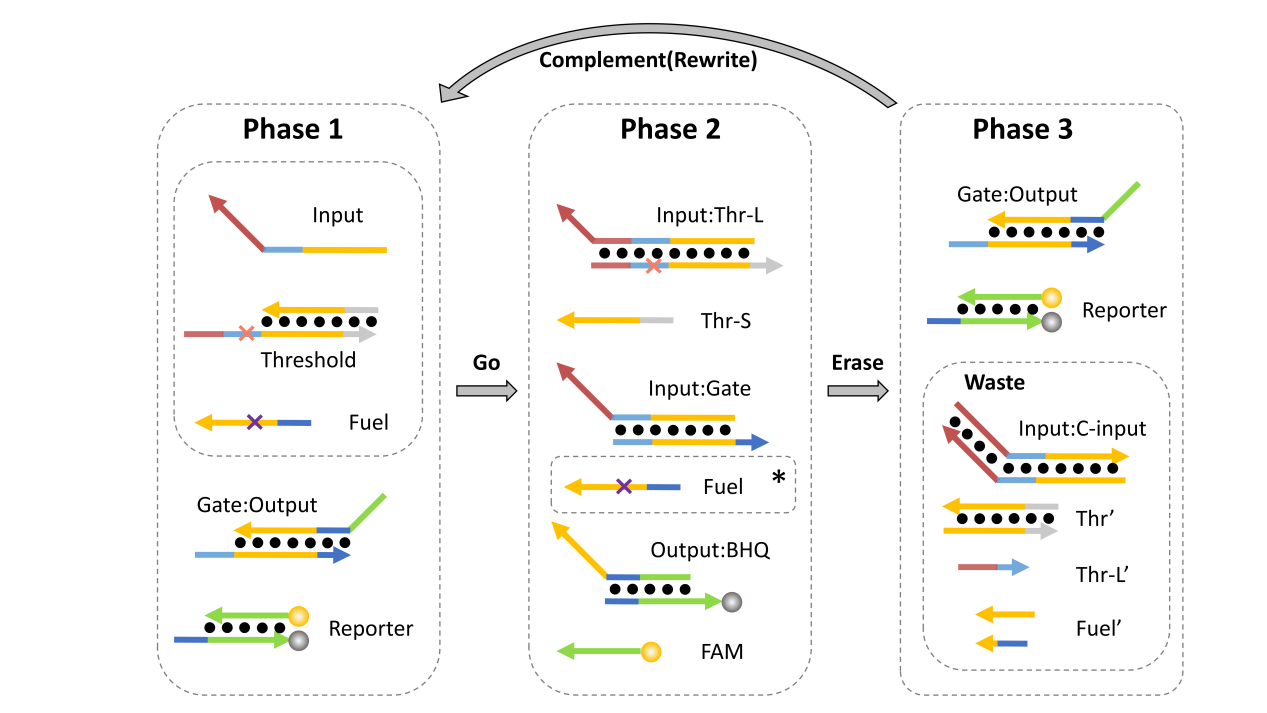


**Figure S1.** Schematic illustration of the detailed states of DNA strands in three phases. The formation of Fuel related double chains in Phase 2 is not shown.


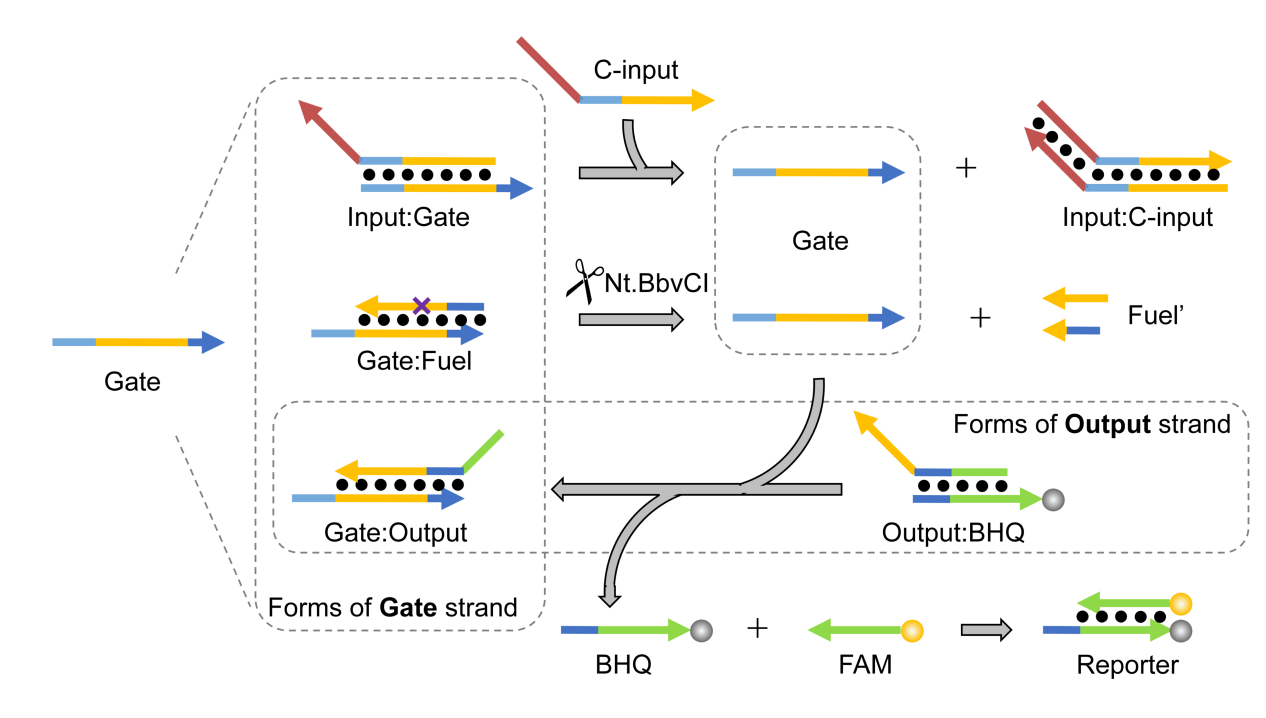


**Figure S2.** Schematic illustration of the recovery of Gate: Output and Reporter in erasure process between Phase 2 and Phase 3.

Focusing on the Gate strand, in Phase 2, the Gate exists in three forms: combined with Input, Fuel, and Output, respectively. After the completion of "erasure" in Phase 3, Gate only forms a duplex with Output. Therefore, Input: Gate and Gate: Fuel in the left box of Figure S2 need to be converted to Gate: Output. In this process, Input: Gate reacts with the added C-input, leading to Gate dissociation. Fuel in the Gate: Fuel is cleaved by the nicking enzyme Nt.BbvCI, forming waste Fuel', and the Gate also dissociates. The dissociated Gate will further react with Output: BHQ, causing Gate to recombine with Output. The generated BHQ-labeled strand will recombine with the FAM-labeled strand and restored the Reporter duplex. The Reporter restoration process will also continually consume free BHQ, allowing the previous step of producing Gate: Output to proceed completely, allowing that all Gates are bound to Output. Consequently, the "skeletal components," Gate: Output and Reporter, have been fully restored, allowing the entire circuit to be reset and reconfigured by re-adding "functional components."


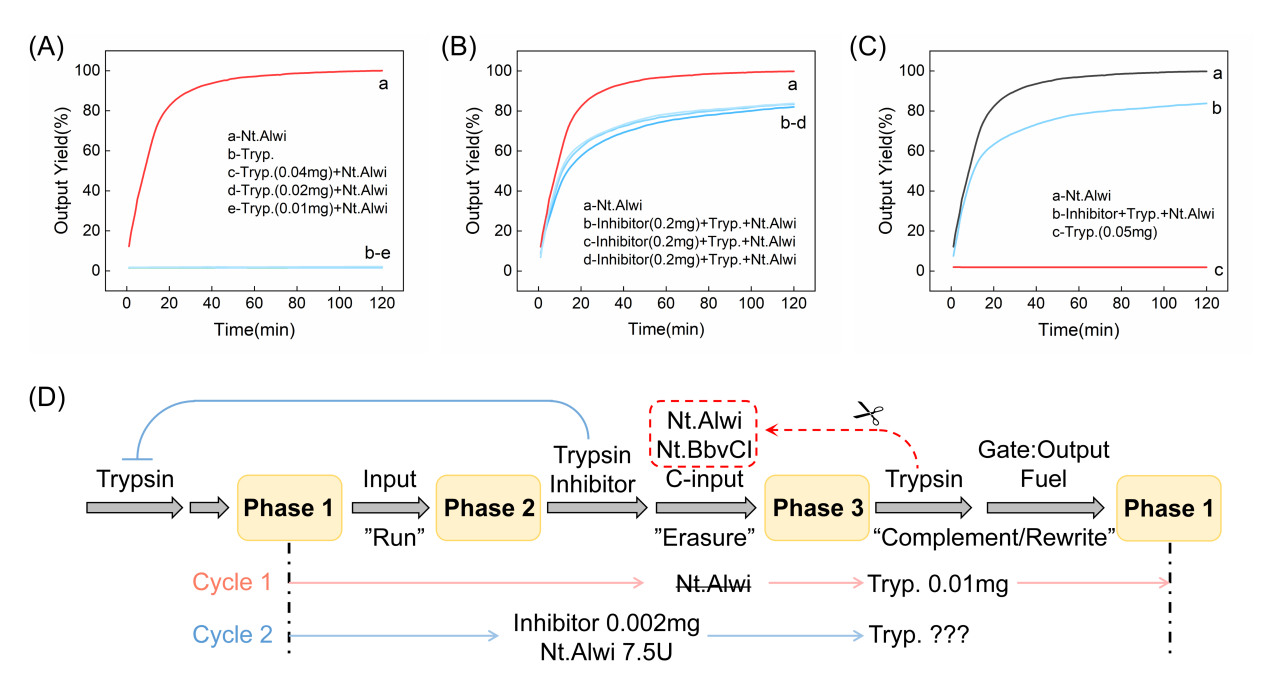


**Figure S3**. Schematic illustration of the enzymes’ function, including nicking enzyme Nt.Alwi, trypsin, and trypsin inhibitor. (A) Fluorescence curves of experiments with various concentrations of trypsin; (B) Fluorescence curves of experiments with various concentrations of trypsin inhibitors; (D) Experimental process and (C) Fluorescence curves of experiments demonstrating the breakthrough of trypsin inhibitors. Ordaining fluorescent results of fluorescently FAM-labeled strand as 100% and Reporter duplex as 0%. The nucleic acid sequence and concentration of this part’s experiments are shown in Table S1.


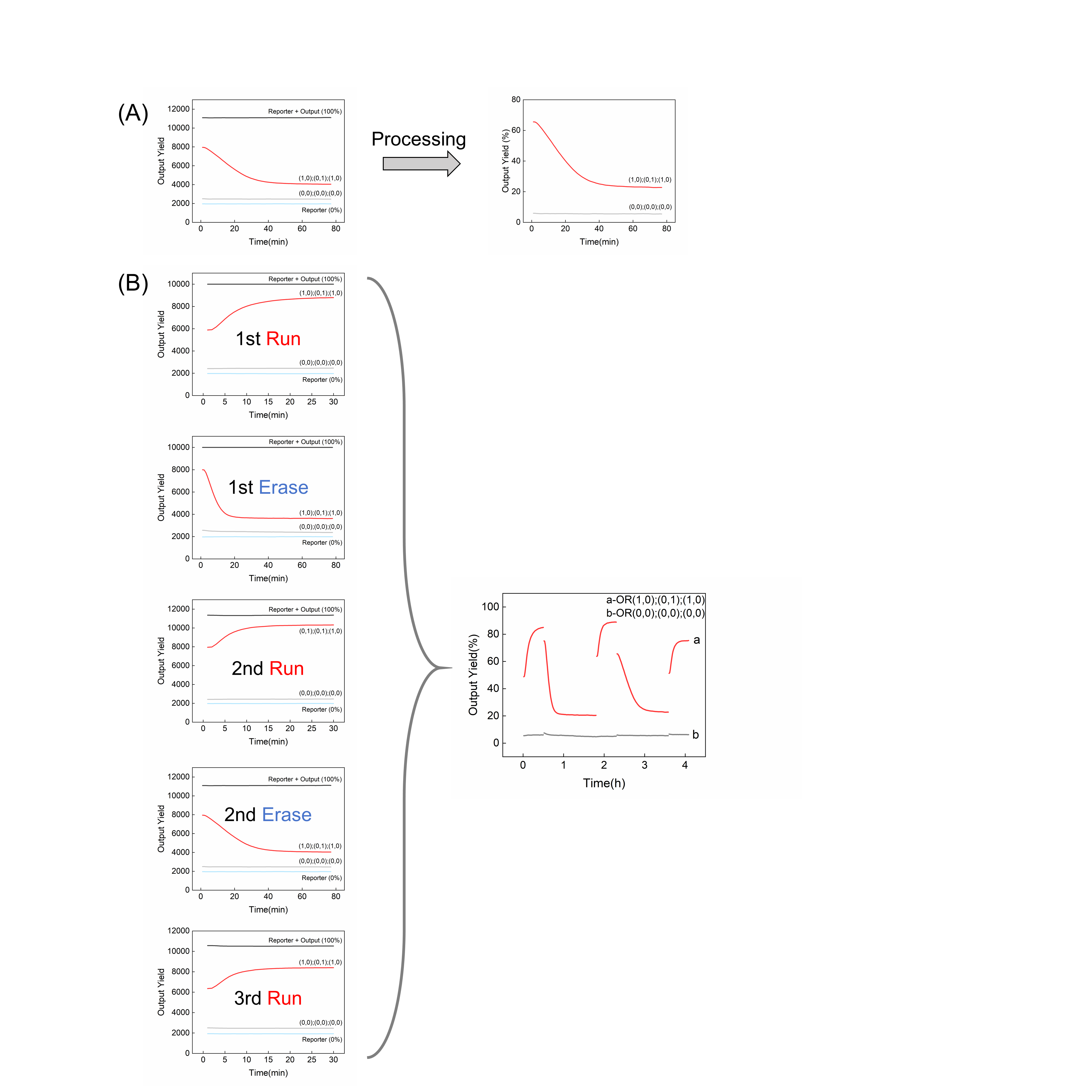


**Figure S4.** Example of processing original fluorescence curves of Simple OR gate. (A) Processing of original fluorescence curves of the second “Erase”; (B) Demonstration of original fluorescence curves for Simple OR gate three-round operations.

**Table S1. Nucleic acid sequences and concentrations used in cutting enzyme, trypsin, and trypsin inhibitor experiments.**

| Name | Sequence (5’—3’) |
| --- | --- |
| FAM | GGTTAAGGATCACCTCAGCAFAM |
| BHQ | BHQTGCTGAGGTGATCCTTAACC |

The FAM concentration in the FAM group is 200μmol/L. Reporters in other groups consist of each 200 μmol/L FAM and BHQ, incubated at 37 ℃.

Table S2. Nucleic acid sequences and concentrations used in the principle validation experiment.

| Name | Sequence (5’—3’) |
| --- | --- |
| 1-FAM | FAMTCTCAAACCTATACA |
| 2-BHQ | GTGGAATGTATAGGTTTGAGABHQ |
| 3-Gate1 | ATGAGGATTGTGGCTGAGGATGGTGGAA |
| 4-Gate2 | GGTGAGTTAGGTGAGGATGTATGAGGA |
| 5-Output1 | ACCTATACATTCCACCATCATCAGCCACAA |
| 6-Output2 | CATCATCAGCCACAATCCTCATAGATCCTCACCTAAC |
| 7-Thr-L | GGATCTATGAGGATTGTGGCTGAGGATGGGTTAG |
| 8-Thr-S | CTAACCCATCATCAGCCACAA |
| 9-Fuel | TTCCACCATCCTCAGCCACAA |
| 10-Input1 | CATCATCAGCCACAATCCTCATAGATCCTCACCTAAC |
| 11-Input2 | TCCTCATAGATCCTCACCTAACTCACCTCTCTCTAAC |
| 12-C-input1 | GTTAGGTGAGGATCTATGAGGATTGTGGCTG |
| 13-C-Input2 | GTTAGAGAGAGGTGAGTTAGGTGAGGATCTAT |

| Experiments | Components | Concentration |
| --- | --- | --- |
| Exploration of "Fuel" concentration | 1-FAM:2-BHQ | 200μM |
|  | 3-Gate:5-Output1 | 200μM |
|  | 7:8 Threshold | 60μM |
|  | 9-Fuel | 0.4/0.6/0.8/1.0mM |
|  | 10-Input1 | 160μM |
| Reuse of simplified OR gate | 1-FAM:2-BHQ | 200μM |
|  | 3-Gate1:5-Output1 | 200μM |
|  | 9-Fuel | 400μM |
|  | 10-Input1 | (1,0) or (0,1) 100μM |
|  | 12-C-input1 | (1,0) or (0,1) 100μM |
| Reuse of complete OR gate | 1-FAM:2-BHQ | 200μM |
|  | 3-Gate1:5-Output1 | 200μM |
|  | 7:8 Threshold | 60μM |
|  | 9-Fuel | 400μM |
|  | 10-Input1 | (1,0) or (0,1) 100μM |
|  | 12-C-input1 | (1,0) or (0,1) 100μM |
| Reuse of YES-OR gate | 1-FAM:2-BHQ | 200μM |
|  | 4-Gate2:6-Output2 | 320μM |
|  | 3-Gate1:5-Output1 | 200μM |
|  | 7:8 Threshold | 60μM |
|  | 9-Fuel | 400μM |
|  | 11-Input2 | (1,1) 250μM  (1,0) or (0,1) 125μM |
|  | 13-C-Input2 | (1,1) 250μM  (1,0) or (0,1) 125μM |
| Function of Nt. Alwi | 1-FAM:2-BHQ | 200μM |
|  | 3-Gate1:5-Output1 | 200μM |
|  | 7:8 Threshold | Figure 3, F(1x = 200μM) |
|  | 9-Fuel | 400μM |
|  | 10-Input1 | Figure 3, F(1x = 200μM) |
|  | 12-C-input1 | Figure 3, F(1x = 200μM) |

**Table S3. Nucleic acid sequences and concentrations used in the T-type input AND gate experiment.**

| Name | Sequence (5’—3’) |
| --- | --- |
| 1-FAM | FAMTCTCAAACCTATACA |
| 2-BHQ | GTGGAATGTATAGGTTTGAGABHQ |
| 3-Gate | ATGAGGATTGTGGCTGAGGATGGTGGAA |
| 4-Gate(9) | ATCTGTTGTTTTATGTTAAGCTGAGGGGAAGTTAAGTGGAA |
| 5-Gate(10) | GATCTGTTGTTTTATGTTAAGCTGAGGGGAAGTTAAGTGGAA |
| 6-Gate(11) | GGATCTGTTGTTTTATGTTAAGCTGAGGGGAAGTTAAGTGGAA |
| 7-Gate(16) | TGAATTGATCTGTTGTTTTATGTTAAGCTGAGGGGAAGTTAAGTGGAA |
| 8-Gate(17) | GTGAATTGATCTGTTGTTTTATGTTAAGCTGAGGGGAAGTTAAGTGGAA |
| 9-Gate(18) | AGTGAATTGATCTGTTGTTTTATGTTAAGCTGAGGGGAAGTTAAGTGGAA |
| 10-Gate(19) | GAGTGAATTGATCTGTTGTTTTATGTTAAGCTGAGGGGAAGTTAAGTGGAA |
| 11-Output | ACCTATACATTCCACCATCATCAGCCACAA |
| 12-Output2 | ACCTATACATTCCACTTAACTTCCCATCAGCTTAACATAAA |
| 13-Thr-L1 | GGATCTATGAGGATTGTGGCTGAGGATGGGTTAG |
| 14-Thr-S1 | CTAACCCATCATCAGCCACAA |
| 15-Thr-L2 | TGAATGGATCTGTTGTTTTATGTTAAGCTGAGGGGAAGTTTGGTTAG |
| 16-Thr-S2 | CTAACCAAACTTCCCATCAGCTTAACATAAA |
| 17-Fuel1 | TTCCACCATCCTCAGCCACAA |
| 18-Fuel2 | TTCCACTTAACTTCCCCTCAGCTTAACATAAA |
| 19-Input1 | TCCTCATAGATCCTCACCTAACTCACCTTCCTTCCAA |
| 20-Input2 | AAACTAAATACTAACAAATCATAAAACAACAGATCCATTCACTCTCAT |
| 21-Input3 | ACTTCCCATCAGCTTATTTTGTTAGTATTTAGTTTTACCATC |
| 22-C-Input2 | ATGAGAGTGAATGGTTCTGTTGTTTTATGATTTGTTAGTATTTAGTTT |
| 23-C-Input3 | GATGGTAAAACTAAATACTAACAAAATAAGCTGATGGGAAGT |

| Experiments | Components | Concentration |
| --- | --- | --- |
| Exploring the length of the T-type input's toe | 1-FAM:2-BHQ | 200μM |
|  | 12-Output2 | 200μM |
|  | 4-Gate(9)  5-Gate(10)  6-Gate(11)  7-Gate(16)  8-Gate(17)  9-Gate(18)  10-Gate(19) | 200μM |
|  | 15:16 Threshold | 100μM |
|  | 18-Fuel2 | 400μM |
|  | 20-Input2 | 200μM |
|  | 21-Input3 | 200μM |
|  | 22-C-Input2 | 200μM |
|  | 23-C-Input3 | 200μM |
| T-AND logic | 1-FAM:2-BHQ | 200μM |
|  | 9-Gate(18):12-Output2 | 200μM |
|  | 15:16 Threshold | 100μM |
|  | 18-Fuel2 | 400μM |
|  | 20-Input2 | (x)=(1) 200μM |
|  | 21-Input3 | (y)=(1) 200μM |
|  | 22-C-Input2 | (x)=(1) 200μM |
|  | 23-C-Input3 | (y)=(1) 200μM |
| Traditional seesaw-AND logic | 1-FAM:2-BHQ | 200μM |
|  | 3-Gate:11-Output | 200μM |
|  | 13:14 Threshold | 1.3/1.5/1.7a |
|  | 17-Fuel1 | 400μM |
|  | 19-Input1 | a=0.4/0.7/1.0x (1x=200μM) |

**Table S4. The nucleic acid sequence and concentration used in OR-AND logic, T-OR logic and AND/OR switching.**

| Name | Sequence (5’—3’) |
| --- | --- |
| 1-FAM | FAMTCTCAAACCTATACA |
| 2-BHQ | GTGGAATGTATAGGTTTGAGABHQ |
| 3-Gate1 | AGTGAATTGATCTGTTGTTTTATGTTAAGCTGAGGGGAAGTTAAGTGGAA |
| 4-Gate2 | CTGATTGAGTGAGCTGAGGATTTAACAAA |
| 5-Output1 | ACCTATACATTCCACTTAACTTCCCATCAGCTTAACATAAA |
| 6-Output2 | ACTTCCCATCAGCTTATTTTGTTAAATCCACAGCTCACTTAA |
| 7-Thr-L1 | TGAATGGATCTGTTGTTTTATGTTAAGCTGAGGGGAAGTTTGGTTAG |
| 8-Thr-S1 | CTAACCAAACTTCCCATCAGCTTAACATAAA |
| 9-Thr-L2 | ATGGGATCTGATTGAGTGAGCTGAGGATTTGAATG |
| 10-Thr-S2 | CATTCAAATCCACAGCTCACT |
| 11-Fuel1 | TTCCACTTAACTTCCCCTCAGCTTAACATAAA |
| 12-Fuel2 | TTTGTTAAATCCTCAGCTCACT |
| 13-Input1  (PM) | ACTTCCCATCAGCTTAACATAAAACAACAGATCCATTCACTCTCAT |
| 14-Input2  (1 mis) | ACTTCCCATCAGCTTATCATAAAACAACAGATCCATTCACTCTCAT |
| 15-Input3  (2 mis) | ACTTCCCATCAGCTTATGATAAAACAACAGATCCATTCACTCTCAT |
| 16-Input4  (3 mis) | ACTTCCCATCAGCTTATGTTAAAACAACAGATCCATTCACTCTCAT |
| 17-Input5  (1 bulb) | ACTTCCCATCAGCTTATTCATAAAACAACAGATCCATTCACTCTCAT |
| 18-Input6  (2 bulbs) | ACTTCCCATCAGCTTATTTCATAAAACAACAGATCCATTCACTCTCAT |
| 19-Input7 | AGCTGTGGATTTAACAAATCATAAAACAACAGATCCATTCACTCTCAT |
| 20-Input8 | AATCCACAGCTCACTCAATCAGATCCCATCCTTCACA |
| 21-C-Input1 | ATGAGAGTGAATGGTTCTGTTGTTTTATGTTAAGCTGATGGGAAGT |
| 22-C-Input2 | ATGAGAGTGAATGGATCTGTTGTTTTATGATAAGCTGATGGGAAGT |
| 23-C-Input3 | ATGAGAGTGAATGGATCTGTTGTTTTATCATAAGCTGATGGGAAGT |
| 24-C-Input4 | ATGAGAGTGAATGGATCTGTTGTTTTAACATAAGCTGATGGGAAGT |
| 25-C-Input5 | ATGAGAGTGAATGGATCTGTTGTTTTATGAATAAGCTGATGGGAAGT |
| 26-C-Input6 | ATGAGAGTGAATGGATCTGTTGTTTTATGAAATAAGCTGATGGGAAGT |
| 27-C-Input7 | ATGAGAGTGAATGGATCTGTTGTTTTATGATTTGTTAAATCCACAGCT |
| 28-C-Input8 | TGTGAAGGATGGGATCTGATTGAGTGAGCTGTGGATT |

| Experiments | Components | Concentration |
| --- | --- | --- |
| Optimization of T-OR-Input | 1-FAM:2-BHQ | 200μM |
|  | 3-Gate1:5-Output1 | 200μM |
|  | 7:8 Threshold | 40μM |
|  | 11-Fuel1 | 400μM |
|  | Input1(PM)  Input2(1mis)  15-Input3(2mis)  16-Input4(3mis)  17-Input5(1bulb)  18-Input6(2bulb) | (1,1) 200μM  (1,0) 100μM |
|  | 21-C-Input1  22-C-Input2  23-C-Input3  24-C-Input4  25-C-Input5  26-C-Input6 | (1,1) 200μM  (1,0) 100μM |
| Reuse of T-OR logic | 1-FAM:2-BHQ | 200μM |
|  | 3-Gate1:5-Output1 | 200μM |
|  | 7:8 Threshold | 40μM |
|  | 11-Fuel1 | 400μM |
|  | 15-Input3(2mis) | (1,1) 200μM  (1,0) 100μM |
|  | 23-C-Input3 | (1,1) 200μM  (1,0) 100μM |
| AND/OR Switch | The same as T-AND and T-OR |  |
| Reuse of OR-AND logic | 1-FAM:2-BHQ | 200μM |
|  | 3-Gate1:5-Output1 | 200μM |
|  | 4-Gate2:6-Output2 | 200μM |
|  | 7:8 Threshold | 100μM |
|  | 9:10 Threshold | 40μM |
|  | 11-Fuel1 | 400μM |
|  | 12-Fuel2 | 400μM |
|  | 19-Input7 | (z)=(1) 200μM |
|  | 20-Input8 | (x,y)=(1,1) 250μM  (x,y)=(1,0) 125μM |
|  | 27-C-Input7 | (z)=(1) 200μM |
|  | 28-C-Input8 | (x,y)=(1,1) 250μM  (x,y)=(1,0) 125μM |
